# Zero Echo Time MRAC on FDG-PET/MR Maintains Diagnostic Accuracy for Alzheimer’s Disease; A Simulation Study Combining ADNI-Data

**DOI:** 10.3389/fnins.2020.569706

**Published:** 2020-11-26

**Authors:** Takahiro Ando, Bradley Kemp, Geoffrey Warnock, Tetsuro Sekine, Sandeep Kaushik, Florian Wiesinger, Gaspar Delso

**Affiliations:** ^1^Department of Radiology, Nippon Medical School, Tokyo, Japan; ^2^Department of Radiology, Mayo Clinic, Rochester, MN, United States; ^3^Institute of Pharmacology and Toxicology, University of Zurich, Zurich, Switzerland; ^4^PMOD Technologies Ltd., Zurich, Switzerland; ^5^Department of Radiology, Nippon Medical School Musashi-Kosugi Hospital, Kawasaki, Japan; ^6^Department of Nuclear Medicine, University Hospital Zurich, Zurich, Switzerland; ^7^GE Healthcare, Waukesha, WI, United States

**Keywords:** PET/MR, attenuation correction, dementia, Alzheimer’s disease, ADNI database, ZTE MRI, atlas-based MRAC, statistical analysis

## Abstract

**Aim:**

Attenuation correction using zero-echo time (ZTE) – magnetic resonance imaging (MRI) (ZTE-MRAC) has become one of the standard methods for brain-positron emission tomography (PET) on commercial PET/MR scanners. Although the accuracy of the net tracer-uptake quantification based on ZTE-MRAC has been validated, that of the diagnosis for dementia has not yet been clarified, especially in terms of automated statistical analysis. The aim of this study was to clarify the impact of ZTE-MRAC on the diagnosis of Alzheimer’s disease (AD) by performing simulation study.

**Methods:**

We recruited 27 subjects, who underwent both PET/computed tomography (CT) and PET/MR (GE SIGNA) examinations. Additionally, we extracted 107 subjects from the Alzheimer Disease Neuroimaging Initiative (ADNI) dataset. From the PET raw data acquired on PET/MR, three FDG-PET series were generated, using two vendor-provided MRAC methods (ZTE and Atlas) and CT-based AC. Following spatial normalization to Montreal Neurological Institute (MNI) space, we calculated each patient’s specific error maps, which correspond to the difference between the PET image corrected using the CTAC method and the PET images corrected using the MRAC methods. To simulate PET maps as if ADNI data had been corrected using MRAC methods, we multiplied each of these 27 error maps with each of the 107 ADNI cases in MNI space. To evaluate the probability of AD in each resulting image, we calculated a cumulative *t*-value using a fully automated method which had been validated not only in the original ADNI dataset but several multi-center studies. In the method, PET score = 1 is the 95% prediction limit of AD. PET score and diagnostic accuracy for the discrimination of AD were evaluated in simulated images using the original ADNI dataset as reference.

**Results:**

Positron emission tomography score was slightly underestimated both in ZTE and Atlas group compared with reference CTAC (−0.0796 ± 0.0938 vs. −0.0784 ± 0.1724). The absolute error of PET score was lower in ZTE than Atlas group (0.098 ± 0.075 vs. 0.145 ± 0.122, *p* < 0.001). A higher correlation to the original PET score was observed in ZTE vs. Atlas group (*R*^2^: 0.982 vs. 0.961). The accuracy for the discrimination of AD patients from normal control was maintained in ZTE and Atlas compared to CTAC (ZTE vs. Atlas. vs. original; 82.5% vs. 82.1% vs. 83.2% (CI 81.8–84.5%), respectively).

**Conclusion:**

For FDG-PET images on PET/MR, attenuation correction using ZTE-MRI had superior accuracy to an atlas-based method in classification for dementia. ZTE maintains the diagnostic accuracy for AD.

## Introduction

Positron emission tomography (PET)/magnetic resonance (MR) has been distributed worldwide and started to be used for the evaluation of dementia both in the clinical and research setting (Drzezga et al., 2014; Barthel et al., 2015; Fendler et al., 2016; Henriksen et al., 2016; Mainta et al., 2017; Zhang et al., 2017; Hope et al., 2019; Prato et al., 2019; Yan et al., 2020). The multi-modal evaluation combining functional images such as PET and morphological images such as MR imaging (MRI) is optimal because each of them provides complementary information (Teipel et al., 2015; Kaltoft et al., 2019). In addition, the motion artifact or partial volume effect on PET images can be minimized by utilizing simultaneous acquisition of MRI (Chen et al., 2018; Yan et al., 2020).

One of the fundamental limitations of PET/MR systems is attenuation correction (AC) derived from MRI (MRAC). With conventional MRI sequences, bone has subtle or no signal intensity because of fast T2^∗^ decay. This results in a difficulty to discriminate bone from other components. This is particularly relevant in brain parenchyma which is covered entirely by the skull. Neglecting attenuation correction from bone causes large and spatially varying error (Andersen et al., 2014).

In this decade, there has already been a vast number of published papers proposing and validating novel MRAC methods. Several novel MRAC methods has been identified that can effectively estimate skull bone (Mehranian et al., 2016; Ladefoged et al., 2017; Teuho et al., 2020). However, the implementation of these methods into commercial PET/MR scanners is still lacking. In addition, the question about how the impact of subtle residual errors on the diagnostic accuracy in dementia is still unanswered, because well-controlled clinical trials using PET/MR scanners have not been conducted so far (Catana et al., 2018; Hope et al., 2019; Teuho et al., 2020). For the evaluation of Alzheimer’s disease (AD), spatial normalization, intensity normalization and statistical analysis such as *t*-value calculation are generally performed (Herholz et al., 2002; Haense et al., 2009). Spatial normalization is performed by non-rigidly transforming the original images to PET template. Intensity normalization consists in dividing the 2-Deoxy-2-[18F] fluoroglucose (FDG) uptake by the average uptake in a reference region (e.g., cerebellum, non-AD-related voxels or whole brain). Following it, *t*-value is statistically calculated as the difference between the patient’s uptake and the average uptake of healthy controls. These steps compensate the variability of brain shape and tracer uptake among subjects. In this setting, not only the net error of tracer uptake, but the distribution of error may impact the result. For example, the underestimation of the reference regions leads to an overestimation of the tracer uptake in AD-related regions, or vice versa. From this point of view, one can argue whether the normal FDG-PET database acquired on PET/computed tomography (CT) scanners could be directly applied to that on PET/MR.

In our previous simulation study, the diagnostic accuracy of FDG-PET on PET/MR was tested by using normal database of FDG-PET on PET/CT (Sekine et al., 2020). That study combined real patients’ FDG-PET images from PET/MR and well-controlled large cohort FDG-PET data from PET/CT in the same space (spatially normalized to the same brain template with the same voxel size). In the study, FDG-PET from PET/MR was generated based on atlas-based MRAC (Atlas) which is one of the commercially available MRAC methods installed in the GE SIGNA PET/MR (Sekine et al., 2016a; Yang et al., 2017a). The results showed that Atlas had similar diagnostic accuracy to the gold-standard, CT-based attenuation correction (CTAC), for the diagnosis of AD, although it slightly impaired sensitivity (Sekine et al., 2020). Currently, Atlas is rarely used for brain PET/MR because the vendor already developed a more accurate MRAC by using zero-echo time MRI (ZTE) which estimates head skull bone by capturing subtle proton density (Delso et al., 2015; Wiesinger et al., 2016). Although several previous studies have clarified that ZTE has substantial accuracy in the net quantification of tracer uptake (i.e., the error is below 10 % generally), the effect of residual error from ZTE was not validated in terms of diagnostic accuracy of AD (Sekine et al., 2016c; Yang et al., 2017b; Sousa et al., 2018; Schramm et al., 2019; Sgard et al., 2019). Before the implementation of ZTE into the clinical FDG-PET/MR evaluation of AD, the diagnostic performance should be validated.

The purpose of this study was to test the diagnostic accuracy of FDG-PET/MR applying vendor-provided ZTE-MRAC to discriminate AD from healthy controls. We performed a simulation study combining real patient’ data and an Alzheimer Disease Neuroimaging Initiative (ADNI) dataset, a well-established large cohort. The whole process was done in an objective and standardized manner, using semi-automatic statistical processing without user interaction.

## Materials and Methods

### Alzheimer Disease Neuroimaging Initiative (ADNI) Data

Data used in the preparation of this article were obtained from the ADNI database^1^. ADNI was launched in 2003 as a public-private partnership, led by Principal Investigator Michael W. Weiner, MD. The primary goal of ADNI has been to test whether serial MRI, PET, other biological markers, and clinical and neuropsychological assessment can be combined to measure the progression of mild cognitive impairment (MCI) and early AD. For up-to-date information^2^.

From ADNI-1 data, we extracted 107 participants (48 healthy and 59 AD participants). The inclusion criteria were completeness of date of birth and diagnosis (healthy or AD) who visit 24 months after 1st PET scan, which inclusion criteria was the same as our previous simulation study focusing on Atlas-based MRAC (Sekine et al., 2020). All raw PET images were of sufficient quality for visual scoring and for software-based analysis using PALZ (PMOD Alzheimer’s Discrimination tool, Zurich, Switzerland). The reported FDG-PET imaging parameters were: injected dose, 185 MBq (5 mCi), dynamic 3D acquisition, six 5-min frames 30–60 min post injection.

### Patients

We recruited 27 patients who underwent both PET/CT and PET/MR for oncologic staging from our previous study (Delso et al., 2018; Sekine et al., 2020). The 27 patients (15 males and 12 females, 60.0 ± 13.0 years) with lymphoma, pheochromocytoma, myeloma, melanoma, Merkel-cell cancer, lung cancer, pancreatic cancer, breast cancer and dementia were collected from another previous study, after excluding 3 patients. Two of these three excluded patients had infarction and one had multiple brain metastases. A neuroradiologist (TS) reviewed and confirmed that all included patients were free of brain abnormalities.

### PET/CT and PET/MR Acquisition

The averaged injected dose of FDG was 534 ± 42 MBq [range, 434–566 MBq]. The PET/CT acquisition followed the standard protocol for a clinical oncology study using a Discovery RX/MI/690/710 PET/CT (GE Healthcare). A helical whole-body CT scan (120 - 140 kV, slice thickness 3.75–5.00 mm, pixel size 1.37 mm× 1.37 mm) was acquired for AC of PET data and diagnostic purposes. Subsequently, a whole-body PET dataset including the head was acquired. Immediately before or after the PET/CT scan, patients were transferred to the integrated PET/MR scanner (SIGNA PET/MR, GE Healthcare), and a brain PET/MR scan was performed as part of the study examination. A 10 min acquisition with a standard head coil (8-channel HD Brain; GE Healthcare) was performed. The duration between tracer injection and PET acquisition was 112 ± 15 min [range, 66–138 min].

During the PET acquisition on the PET/MR, liver acquisition with volume acceleration flex (LAVA-Flex) T1w images (axial acquisition, TR ∼ 4 ms, TE 2.23 ms, flip angle 5°, slice thickness 5.2 mm with 2.6 mm overlap, 120 slices, pixel size 1.95 mm × 1.95 mm, number of excitations (NEX) 0.9, acquisition time: 18 s) were acquired for vendor-provided atlas-based AC.

Additionally, proton-density ZTE MR images (sagittal acquisition; non-selective hard pulse excitation; 3-dimensional center-out radial acquisition; repetition time, 410 ms; nominal echo time, 0 ms; transmit-receive switching times, 20 μs; flip angle, 1°; slice thickness, 2.78 mm; 118 slices; pixel size, 1.17 mm × 1.17 mm; bandwidth ± 62.5 kHz; number of excitations, 4; acquisition time, 48 s; spokes per segment, 512) were acquired.

### Attenuation Map Generation

For each patient, 3 AC maps were generated, Atlas-AC, ZTE-AC and CT-AC. The brief overview of the algorithm was described below.

### Attenuation MAP Based on Atlas Methods (Sekine et al., 2016a; Yang et al., 2017a)

An atlas-based method was used to derive a pseudo-CT that included continuous attenuation information for the head, using a single-head atlas, which was provided by the vendor and is based on CT images from 50 subjects. The pseudo-CT was generated as follows. First, 3-mm Hessian-bone enhancement from LAVA in-phase images was performed. Second, pseudo-CT was generated by rigid and non-rigid B-spine-based elastic registration between bone-enhanced MR image and the head atlas. Third, the attenuation map is generated from the pseudo-CT using the standard energy conversion and resampling. Finally, the MR hardware, coil, and bed are added to the attenuation map.

### Attenuation MAP Based on ZTE Imaging (Sekine et al., 2016c; Wiesinger et al., 2016; Yang et al., 2017b)

The processing steps detailed below were performed using custom Matlab scripts (version 7.11.0; The MathWorks) but whole steps are the same as the commercial version. Attenuation Map based on ZTE imaging includes three steps. First, bias correction was applied. Second, tissue classification was performed by thresholding for soft tissue/bone and bone/air, based on the values of the tissue and air histogram peaks. Third, continuous attenuation values were assigned to the bone, based on the linear correlation between CT values and ZTE MR values (offset, 300; slope, 2,400; maximum bone value, 2,000 Hounsfield units). To the soft tissue, a fixed attenuation value of 42 Hounsfield units was assigned. The formulas to generate the thresholds and attenuation value were defined empirically before the study and remained constant for all patients.

### Co-registered Attenuation Map Based on CT Method

The processing steps detailed below were performed using custom Matlab scripts and PMOD (version 4.0; PMOD Inc., Zurich, Switzerland). The co-registered CT-AC map was generated as follows. First, the original head CT was exported from the PET/CT scanner and converted into AC-map using a Matlab version of the same bilinear mapping implemented in the SIGNA PET/MRI. Second, from this map, the CT table was removed manually. Third, a threshold was set to extract the outside air component from the CT-AC map. None of the images used in this study contained artifacts likely to affect air thresholding. Fourth, a normalized mutual information matching algorithm (PMOD) was used to derive the registration parameters necessary to match CT to LAVA-Flex T1w, and the final matching was performed using custom Matlab routines. Finally, the CT-AC map was superimposed on the atlas-AC map, thereby replacing it.

### Reconstruction of PET Images

Only the raw PET data from the TOF PET/MR examination were used. PET images were reconstructed with AC based on each of the 3 attenuation maps and the following parameters: fully 3-dimensional ordered-subset expectation maximization iterative reconstruction; subsets, 28; iterations, 8; pixel size, 1.17 mm × 1.17 mm; point spread function modeling; transaxial post reconstruction gaussian filter cutoff, 3 mm; axial filter, 1:4:1; scatter; normalization; dead-time and decay corrections; TOF reconstruction.

### Automated Software for AD Probability Assessment

Automated AD probability assessment was performed in a commercially available tool which was established in the ADNI study and validated in several multi-center study, such as NEST-DD and SEADS-JAPAN (PMOD Alzheimer’s Discrimination, PALZ) (Haense et al., 2009; Herholz et al., 2011; Ito et al., 2015). The software ran the following procedure, in a fully automated workflow based on the previous study (Herholz et al., 2002). First, spatial normalization is performed by transforming the original images to the SPM 99 PET template, followed by smoothing with Gaussian filter of 12 × 12 × 12 mm. In these images, voxel values are normalized by dividing each image voxel value by the mean voxel value, averaged within a mask representing voxels in which FDG uptake is typically preserved even in AD patients. The expected value in each voxel is calculated from a pre-stored, age-matched, reference PET database of healthy controls using voxel-wise age regression parameters. By comparing the voxel-wise differences between expected value and the patient-specific value, a Student’s *t*-value is calculated. The AD t-sum is calculated by summing the *t*-value in predefined AD-related voxels. Finally, the PET Score was calculated as log2 (AD t-sum/11089 +1), for which the 95% prediction limit (11089) of AD t-sum was established in the ADNI or NEST-DD multi-center trial. This analysis was initially performed in all 107 ADNI-PET data (e.g., *PETscore ^*original*^*) before multiplication with the 27 error maps.

### Creation of Simulated Data: ADNI-Data With Atlas-AC or ZTE-AC

The whole steps were done according to the previous report (Sekine et al., 2020). All simulation steps were performed in MNI space with same spatial resolution (2 mm isotropic voxels). First, we divided the locally acquired PET images based on atlas AC or ZTE by those based on CTAC (27 patients) (e.g., _*Error*_*PET*^*pt*−*i*^). Second, the resulting images were spatially normalized to the SPM99 PET template using the transformation calculated for PET images based on CTAC to the template, then a Gaussian filter of 12 × 12 × 12 mm full-width half-maximum was applied (${}_{E\,r\,r\,o\,r}^{N\,o\,r\,m}\,P\,E\,T^{p\,t-i}$). A brain mask was applied to avoid distortion at the edges of the measured data. These steps were designed to replicate the preprocessing steps used in the PMOD Alzheimer’s Discrimination tool, as used to calculate PET score. Therefore, the resulting images were the error maps (between MRAC and CTAC) in the same image space as the spatially normalized ADNI PET data (__  ^Norm PET_ADNI–*j* ^ _). It minimized the error derived from the difference of scan condition (e.g., imaging protocol of PET scanner) between ADNI data and patients’ data. Third, we multiplied each of the 107 normalized ADNI data with each of the 27 normalized error maps, resulting in 107 × 27 = 2889 normalized PET images (e.g., ${}_{E\,r\,r\,o\,r}^{N\,o\,r\,m}\,P\,E\,T_{A\,D\,N\,I-j}^{p\,t-i}$) for each MRAC method. Thus, the value-error was simply imposed in a voxel-wise manner and further PET score calculation was performed without additional need for spatial deformation or filtering. Therefore, we expected any bias due to impaired spatial normalization or differences in PET acquisition protocol to be minimized. For each of these 5778 images (2889 × 2), PALZ analysis was performed to calculate the PET score based on MRAC (*PETscore*^*MRAC*^).

### Evaluation of Diagnostic Accuracy for Alzheimer’s Disease

We calculated the absolute PET score difference between *PETscore*^*MRAC*^ and *PETscore*^*original*^. This PET score difference was compared between Atlas-AC and ZTE-AC by using paired *t*-test. We also drew performed regression and Bland-Altman analysis between *PETscore*^*MRAC*^ and *PETscore*^*original*^.

We evaluated the diagnostic accuracy of discrimination of AD from normal patients. Setting cut-off value to PET score = 1 based on a previous study, we calculated the accuracy, sensitivity, and specificity of each MRAC series (Haense et al., 2009; Herholz et al., 2011). Additionally, we performed a receiver-operating-curve analysis to define the modified cut-off values for each MRAC series according to the maximum value of the Youden index (Youden, 1950; Schisterman et al., 2005).

## Results

**FIGURE 1:**
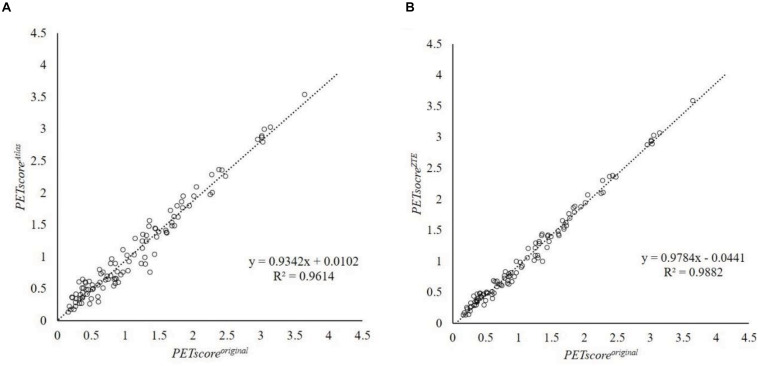
Regression line analysis between *PETscore*^*MRAC*^ and *PETscore^*original*^.* This plot shows 107 data which are averaged from 27 error maps. The horizontal axis shows *PETscore*^*original*^ and the vertical axis shows *PETscore*^*Atlas*^ **(A)** or *PETscore*^*ZTE*^ **(B)**. Regression equation were *y* = 0.9342x + 0.0102 **(A)** and *y* = 0.9784x + 0.0441 **(B)**, respectively. *R*^2^ were 0.9614 **(A)** and 0.9882 **(B)**, respectively. R^2^, coefficient of determination.

*PETscore*^*Atlas*^ and *PETscore*^*ZTE*^ were underestimated compared to *PETscore*^*original*^ (*PETscore*^*Atlas*^ minus *PETscore^*original*^*: −0.0784 ± 0.1724; *PETscore*^*ZTE*^ minus *PETscore*^*original*^: −0.0796 ± 0.0938) (Tables 1A,B and Figure 1). The absolute PETscore difference between *PETscore*^*ZTE*^ and *PETscore*^*original*^ was slightly lower than that between *PETscore*^*Atlas*^ and *PETscore*^*original*^ (0.098 ± 0.075 vs. 0.145 ± 0.122, *p* < 0.0001) (Table 1C).

**TABLE 1-A:** *PETscore* in each 2889 simulated dataset.

*PETscore*	Ave	SD	Max	Min
*PETscore*^*Atlas*^	1.0792	0.7614	3.6136	0.0956
*PETscore*^*ZTE*^	1.0755	0.7852	3.6193	0.1118
*PETscore*^*original*^	1.1443	0.7972	3.6488	0.1669

*Ave, average; SD, standard deviation; Max, maximum; Min, minimum.*

**TABLE 1-B:** Positron emission tomography score difference (*PETscore*^*MRAC*^-*PETscore*^*original*^) in each 2889 simulated dataset.

PET score difference	Ave	SD	Max	Min
Atlas	−0.0651	0.1669	0.3840	−0.8404
ZTE	−0.0689	0.0918	0.2070	−0.4873

*Ave, average; SD, standard deviation; Max, maximum; Min, minimum.*

**TABLE 1-C:** Absolute PET score difference (|*PETscore*^*MRAC*^-*PETscore*^*original*^|) in each 2889 simulated dataset.

Absolute PET score difference	Ave	SD	Max	Min	*P*-value
Atlas	0.1392	0.1128	0.8404	0.0000	< 0.0001
ZTE	0.0885	0.0731	0.4873	0.0000	

*Paired samples *t*-test was performed between |*PETscore*^*Atlas*^-*PETscore*^*original*^| and |*PETscore*^*ZTE*^-*PETscore*^*original*^|. Ave, average; SD, standard deviation; Max, maximum; Min, minimum.*

**FIGURE 2:**
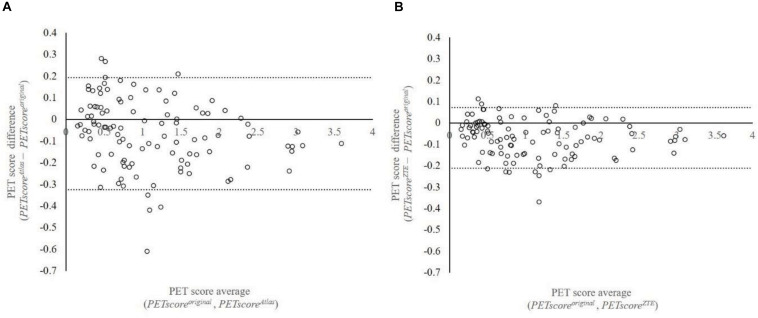
Bland-Altman plot between *PETscore*^*MRAC*^ and *PETscore*^*original*^. This plot shows 107 data which are averaged based on 27 error maps. The vertical axis shows difference of *PETscore*^*original*^ and *PETscore*^*Atlas*^ **(A)** or *PETscore*^*ZTE*^ **(B)**. The horizontal axis shows average of *PETscore*^*original*^ and *PETscore*^*Atlas*^ **(A)** or *PETscore*^*ZTE*^ **(B)**. LOA were –0.323 to 0.194 **(A)** and –0.211 to 0.073 **(B)**, respectively. LOA, limits of agreement.

**FIGURE 3:**
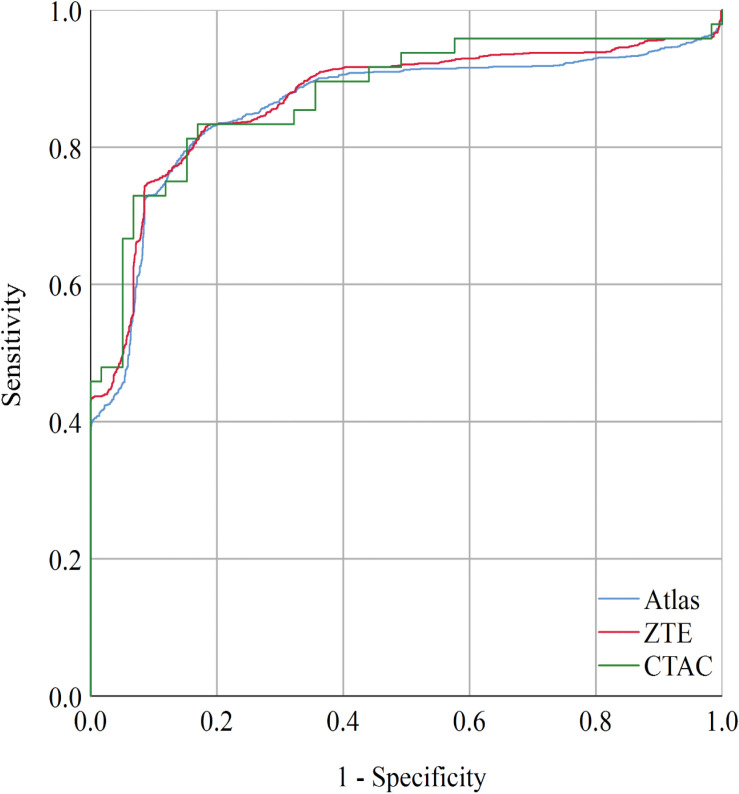
When setting the cut-off value to *PETscore*^*ZTE*^ = 1.143, the sensitivity and specificity were 75 and 91% with AUC = 0.870 (CI: 0.855–0.884). When setting the cut-off value to *PETscore*^*Atlas*^ = 0.924, the sensitivity and specificity were 81 and 84% with AUC = 0.859 (CI: 0.844–0.875). The AUC of CTAC was 0.876 (CI: 0.862–0.890). AUC, area under curve.

Regression and Bland-Altman analysis between *PETscore*^*original*^ and either *PETscore*^*Atlas*^ or *PETscore*^*ZTE*^ are shown in Figures 2, 3. The slope of the regression lines between *PETscore*^*original*^ and *PETscore*^*Atlas*^ were 0.9342 and between *PETscore*^*original*^ and *PETscore*^*ZTE*^ 0.9882. The coefficient of determination (*R*^2^) was higher for *PETscore*^*ZTE*^ and limits of agreement (LOA) of *PETscore*^*ZTE*^ was lower than *PETscore*^*Atlas*^ (*R*^2^: 0.982 vs. 0.961, LOA: −0.211 to 0.073 vs. −0.323 to 0.194).

Accuracy, sensitivity, and specificity of each attenuation method with cut-off values of PET score = 1 are shown in Table 2. The accuracy for the discrimination of AD patients from normal control derived from Atlas or ZTE was maintained [Atlas vs. ZTE vs. original; 82.5% (CI 81.0–83.8%) vs. 82.1% (CI 80.7–83.5%) vs. 83.2% (CI 81.8–84.5%)] but sensitivity was slightly impaired compared with those derived from CTAC [Atlas vs. ZTE vs. original; 77.2% (CI 74.9–79.5%) vs. 78.6% (76.3–80.8%), 83.3% (CI 81.2–85.3%)].

**TABLE 2:** Diagnostic accuracy of original PET data and simulated MRAC PET data with various PET score values in each of the 2889 simulated datasets.

	Cut-off value	Accuracy	Sensitivity	Specificity
CTAC	1	83.2% (CI 81.8–84.5%)	83.3% (CI 81.2–85.3%)	83.1% (CI 81.1–84.9%)
Atlas-AC	1	82.5% (CI 81.0–83.8%)	77.2% (CI 74.9–79.5%)	86.7% (CI 84.9–88.3%)
Atlas-AC	0.924	82.5% (CI 81.0–83.8%)	80.8% (CI 78.5–82.9%)	83.8% (CI 81.9–85.6%)
ZTE-AC	1	82.1% (CI 80.7–83.5%)	78.6% (CI 76.3–80.8%)	85.0% (CI 83.1–86.7%)
ZTE-AC	1.143	83.7% (CI 82.3–85.1%)	74.8% (CI 72.3–77.1%)	91.0% (CI 89.5–92.4%)

The receiver-operating-curve analysis to determine modified cut-off values of PET score is shown in Figure 3. The modified cut off values are *PETscore*^*Atlas*^ = 0.924, *PETscore*^*ZTE*^ = 1.143, respectively. Setting cut-off values to these modified scores, the accuracy and specificity of ZTE was slightly improved [83.7% (CI 82.3–85.1%) and 91.0% (CI 89.5–92.4%)]. The values of area under the curve (AUC) of ROC curve were not impaired in Atlas {0.859 [CI: 0.844–0.875]) or ZTE (0.870 [CI: 0.855–0.884]), compared with CTAC (0.876 [CI: 0.862–0.890])}.

## Discussion

In the current study, we validated the diagnostic performance of two types of MRAC methods in the classification of AD with FDG-PET. We generated simulated images by multiplying well-controlled cohort, ADNI-dataset and real patients’ error maps derived from each MRAC method. The results show the PETscore based on both Atlas and ZTE were underestimated compared with the original PETscore based on CTAC. ZTE had smaller variability of the error than Atlas MRAC. The accuracy was not impaired by either MRAC, though the sensitivity was slightly impaired due to the underestimation of PETscore. By determining and setting modified cut off values, slightly better accuracy and specificity of Atlas and ZTE were obtained.

There are several studies which compared the accuracy of Atlas and ZTE MRAC (Sekine et al., 2016c; Yang et al., 2017b; Sousa et al., 2018; Sgard et al., 2019). These studies revealed that both the averaged error and the variability (standard deviation) of the error among subjects were minimized by ZTE compared to Atlas. Our results are in agreement with these previous studies: both averaged and standard deviation of absolute PET score difference were lower in ZTE than in Atlas. In addition, there were two interesting results observed. First, the averaged PET score difference of ZTE was not superior to Atlas. This may indicate that the minimization of the net regional error doesn’t directly lead to an improvement of the diagnosis accuracy of dementia after the value normalization and t-score calculation. Second, the PETscore error was not systematically under- or over-estimated in each of the original 107 datasets. The t-score calculation is based on the difference from averages and the magnitude of the standard deviation in each region derived from a normal database. It resulted in heterogeneous effect on each 107 dataset by identical MRAC error.

In one sole previous study, statistical analysis using SPM procedure was performed for FDG-PET generated based on ZTE. Sgard et al. (2019) recruited 50 patients who underwent FDG-PET/MR as part of an investigation of suspected dementia. The study revealed that ZTE was more accurate than Atlas for the quantification of FDG uptake especially in the parietotemporal junction, one of the earliest regions involved in AD. In that study, the relative difference both of net SUV value and of t-score calculated by using SPM was evaluated. Interestingly, the distribution is similar but not the same between the two. For example, supratentorial regions were generally underestimated by ZTE MRAC but this underestimation was not observed after the SPM procedure because the procedure includes intensity normalization. Underestimation in the reference regions leads to overestimation, which compensates the underestimation in the target regions. In addition, they evaluated voxel-wise differences between normal and abnormal brain FDG-PET. Although ZTE-MRAC was more accurate than Atlas-AC, there were still clear differences between CT-AC and ZTE-AC. One can expect that even if the regional accuracy in AD-related voxels is achieved by any MRAC, the result after value-normalization and *t*-value calculation should be validated separately.

In terms of the validation of MRAC, one of the largest studies is the two-institutional study conducted by Ladefoged et al. (2017). They performed cross-comparison of 11 MRAC methods to 337 subjects undergoing brain PET/MR and CT. They clarified all of the novel methods provided by each research group had an acceptable error (e.g., the MRAC error to silver standard CTAC is less than 5 %). Though one of these methods is freely accessible via web-based process^3^, MRAC which is not supported by vendors is difficult to implement into clinical workflow (Burgos et al., 2015; Sekine et al., 2016b). Apart from these atlas/template-based or segmentation-based MRAC methods, deep learning-based MRAC have been proposed in research field. In published data, the method seems to be more accurate than clinically available methods such as Atlas-AC or ZTE-AC (Liu et al., 2018; Arabi et al., 2019; Shiri et al., 2019). However, there is some variability of the methodology in each published paper. Thus, the clinical implementation of this MRAC method is not achieved yet.

The diagnostic accuracy was evaluated by using a single software tool, and not done by other statistical approaches or visual assessment. It was one of main limitations of the current study. However, the diagnostic concept was similar to that used in other software and in visual inspection. These evaluations generally apply value-normalization and *t*-/*z*-score calculation. We chose this software because of four reasons. First, the whole steps were done in semi-automatic manner without any interruption. It was practical and assured objective results. Second, the model had validated in several large cohort study. As a result, the method is used widely in clinical setting as commercial software. Third, the statistical model is the simplest to minimize the secondary effect by the statistical model. The novel classification method has additional steps such as partial volume effect and the normalization combining T1WI (Samper-González et al., 2018). However, in the complicated statistical mode, it becomes difficult to separate the primary effect of MRAC error to value-normalization from whole effect of MRAC error as the summing of each analysis steps.

The limitations of this study were as below. First, the conclusions of the current study were based on a simulation study rather than on “real” PET images of AD subjects reconstructed with both MR and CT AC. To confirm the result in the current study, well-controlled clinical trials using PET/MR scanners should be performed in the future. Second, some of the error maps were generated from patients imaged for the evaluation of malignancy. The error derived from MRAC was mainly derived from the incorrect estimation of skull thickness/density which does not differ between oncology and dementia patients. Third, we only recruited a limited number of patients, *n* = 27 for the generation of MRAC. The simulation model multiplying 27 error maps for each ADNI data might cause statistical ambiguousness. However, a significant improvement of absolute PETscore based on ZTE was observed. The validation of MRAC on large cohorts is difficult to perform because additional CT is required to acquire the gold-standard AC map.

## Conclusion

In conclusion, statistical analysis utilizing a normal database scanned by PET/CT can be applied to FDG-PET images scanned by PET/MR implementing ZTE-MRAC. ZTE-MRAC had superior accuracy to Atlas-MRAC even after statistical analysis such as value-normalization and *t*-value calculation. It is expected that the diagnostic accuracy for the discrimination of AD from healthy control would be maintained by using FDG-PET scanned by commercial PET/MR system.

## Data Availability Statement

The raw data supporting the conclusions of this article will be made available by the authors, without undue reservation.

## Ethics Statement

The studies involving human participants were reviewed and approved by the Local Institutional Review Board of Mayo Clinic. The patients/participants provided their written informed consent to participate in this study.

## Author Contributions

TA, TS, and GD designed the study and contributed to the data analysis. BK, SK, FW, and GD contributed to the data acquisition. SK, FW, and GD generated PET images. GW advised the data analysis. TA, GW, TS, and GD contributed to drafting of the manuscript together. All authors contributed to the article and approved the submitted version.

## Conflict of Interest

GD, SK, and FW are employees of GE Healthcare. Only non-GE employees had control of inclusion of data and information that might present a conflict of interest for authors who are employees of GE Healthcare. GW is an employee of PMOD Technologies LLC. The remaining authors declare that the research was conducted in the absence of any commercial or financial relationships that could be construed as a potential conflict of interest.

## Footnotes

1 adni.loni.usc.edu

2 www.adni-info.org

3 http://niftyweb.cs.ucl.ac.uk/program.php?p=PCT
